# Editorial: Multimodal and Integrative Analysis of Single-Cell or Bulk Sequencing Data

**DOI:** 10.3389/fgene.2021.658185

**Published:** 2021-02-26

**Authors:** Geng Chen

**Affiliations:** ^1^Center for Bioinformatics and Computational Biology, Shanghai Key Laboratory of Regulatory Biology, Institute of Biomedical Sciences, School of Life Sciences, East China Normal University, Shanghai, China; ^2^Genecast Biotechnology Co., Ltd., Wuxi, China

**Keywords:** multi-omics, integrative analysis, single-cell omics, multi-omics analysis, next-generation sequencing

Biological systems often involve the complex interactions among the molecules from different omics layers, including genome, methylome, transcriptome, proteome, metabolome, and even microbiome. At the genome level, diverse types of variants (e.g., single nucleotide variations, small insertions or deletions, and structural variations) that could be associated with a wide range of phenotypes or diseases may occur on the genome. For epigenome, it includes a variety of epigenetic modifications, such as covalent modifications on DNA and histones, chromatin accessibility and compaction, as well as the higher-order conformation of chromosome domains, which form an intricate regulatory network that can influence the chromatin structure and gene expression (Weinhold, 2006; Allis and Jenuwein, 2016). Exploration of the transcriptome was greatly revolutionized by RNA-seq technologies, which have gradually replaced traditional microarrays and provided unprecedented insights into the dynamics and complexity of gene expression (Costa et al., 2010; Stark et al., 2019). Specifically, many long non-coding RNAs (lncRNAs) and circular RNAs (circRNAs) were found to have critical regulatory functions in diverse biological processes (Marchese et al., 2017; Xiao et al., 2020). Proteins encoded by mRNAs are generally organized into higher-order structures and networks to perform catalytic, synthetic, and regulatory functions at specific times and locations (Aebersold and Mann, 2016). Mass spectrometry (MS)-based methods [such as liquid chromatography-MS/MS (LC-MS/MS)] greatly revolutionized proteome profiling and largely facilitated the dissection of complex biological processes and phenotypes (Angel et al., 2012). Furthermore, metabolome can theoretically link the genome, transcriptome, and proteome to phenotype (Misra et al., 2018). The levels and relative ratios of metabolites could generally reflect the metabolic functions, thus abnormal perturbations that beyond the normal range may indicate certain diseases (Hasin et al., 2017). Additionally, microbiomes may also significantly contribute to the biology and diverse phenotypes of their partner organisms, which can reveal the interactions between the genome and environment of the host organism (Knight et al., 2017; Lynch and Hsiao, 2019). Therefore, multi-omics analysis can promote the development of systems biology, which is essential for comprehensively investigating the dynamic changes and interactions of cellular molecules as well as understanding the underlying mechanisms (Figure 1).

**Figure 1:**
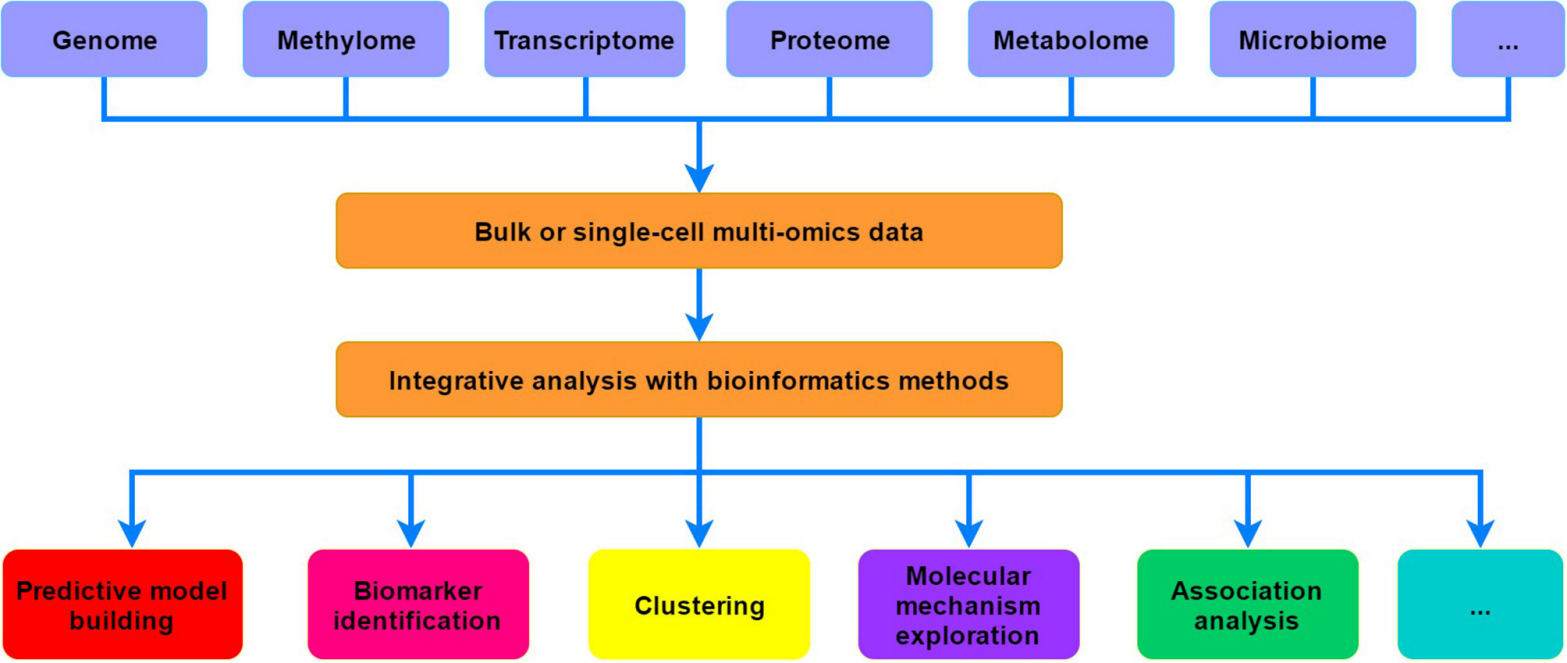
A simple schematic view of systems biology researches based on multi-omics data.

In this research topic, a number of research teams conducted integrative analyses to explore the molecular mechanisms or identify potential biomarkers for certain diseases/disorders. Chen et al. systematically investigated the expression profiles of placenta accreta spectrum (PAS) at both transcriptomic and proteomic levels, which provided novel insights into the underlying molecular mechanism of PAS. Through joint analysis of the interaction networks among miRNAs, mRNAs, and lncRNAs, Wang et al. identified novel potential prognostic markers for luminal breast cancer patients. Sun et al. revealed that HIF-1α pathway-related lncRNA-HEIPP (high expression in preeclampsia placenta) could play an important role in the pathogenesis of preeclampsia based on the multi-omics exploration. Wang et al. performed an integrative analysis of the underlying mechanisms of noise-induced hearing loss (NIHL) and suggested that the inflammatory pathways are closely associated with the auditory organ changes of NIHL. Zhang et al. found that G-quadruplexes could be potential targets for the drug discovery of severe acute respiratory syndrome coronavirus 2 (SARS-CoV-2) by systematically analyzing the non-canonical secondary structures of G-quadruplexes in both positive and negative-sense strands of SARS-CoV-2. Wang et al. revealed that the expression profile of IGFBP7 could be a potential biomarker for vasculature in response to traumatic brain injury and TGFβ signaling might be closely correlated to the upregulation of IGFBP7. Yuan et al. systematically explored the expression patterns of major depressive disorder (MDD) and uncovered that Ephrin signaling and Ras protein signal transduction could be associated with the MDD pathogenesis. Wang et al. identified potential diagnostic and prognostic biomarkers for colorectal cancer based on an integrative analysis of the datasets from different public databases. Moreover, Xiong et al. developed an integrative computational approach of ASDmiR for identifying the potential pathogenic genes, networks, and modules correlated with autism spectrum disorder. Overall, these studies performed joint analyses on the data from distinct omics layers, which gained novel insights into different diseases.

Although the development and innovation of single-cell approaches will gradually lead to a shift from bulk integrative analysis to a detailed exploration of individual cells, bulk strategies are complementary with single-cell approaches to get whole-system and cell-based perspectives and mechanisms for health and disease. One main limitation of bulk profiling methods is that they cannot accurately disentangle the cellular heterogeneity, thus single-cell exploration is essential for better elucidating the cellular behaviors and cell-to-cell variations for both basic and clinical research. However, compared to conventional bulk approaches, the single-cell technologies currently available for distinct omics are still in the early stages of development, which are often with relatively lower capture efficiency and higher technical noise (Chen et al., 2019). The improvement of the experimental procedures for single-cell protocols will reduce the technical noise and sparsity of multi-omics data, and increase the sensitivity and specificity of multimodal dissection. Moreover, conducting the single-cell study on a large number of samples is still expensive and time-consuming, the decreasing cost and simplified operation of single-cell profiling will make the multi-omics analysis more affordable and practicable. Since bulk strategies are feasible to study large-scale samples, combing bulk and single-cell data with deconvolution methods could be a good solution to investigate a multitude of individuals in a cell-type-specific manner (Li et al., 2020). Besides, the computational methods for joint analysis of single-cell multimodal data are just emerging in recent years, novel bioinformatics tools are required to more efficiently integrate single-cell multi-omics data. On the other hand, existing bulk and single-cell sequencing protocols are mainly based on next-generation sequencing technologies. We envision that the utilization of third-generation sequencing approaches [e.g., Nanopore (Garalde et al., 2018)] to produce super long reads in the bulk or single-cell omics studies will largely benefit the downstream data analysis and facilitate the development of systems biology.

## Author Contributions

GC conceived and wrote the manuscript.

## Conflict of Interest

GC was employed by company Genecast Biotechnology Co., Ltd.
